# Human Skin Aging: Physiology and Pathophysiology

**DOI:** 10.3390/biology15161407

**Published:** 2026-08-17

**Authors:** Vu Thi Phuong Thao, Nguyen Thi Phan Thuy

**Affiliations:** Ho Chi Minh City Hospital of Dermato-Venereology, Ho Chi Minh City 70000, Vietnam; bsphanthuy@yahoo.com

**Keywords:** skin aging, intrinsic aging, extrinsic aging, geriatric skin disease

## Abstract

Skin is the outermost layer which covers all organs of the body. Skin can be divided into three layers, including epidermis, dermis and hypodermis. Similarly to many organs of the body, skin also experiences biological process that could not resist aging. There are the following two types of human skin aging: endogenous and exogenous. Endogenous aging involves a series of physiological changes that gradually occur over time and are influenced by genes and hormones. In contrast, extrinsic aging (or exogenous aging), also known as photoaging, involves significant structural and functional changes due to external factors, mainly due to lack of sun protection, smoking, air pollution, lifestyle, etc. Skin aging not only affects the appearance but also makes the skin more sensitive to geriatric skin diseases. In order to treat geriatric skin disease effectively and appropriately, it is crucial to study skin aging from a clinical and histological perspective.

## Simple Summary

Skin aging is a natural phenomenon which can be affected by intrinsic factors (hormones, gene mutations, and reactive oxygen species) and extrinsic factors (solar radiation, pollution, etc.). Changes in three layers and appendages of the skin result in the appearance of wrinkles, dehydration, decrease elasticity, hair loss, reduced nail growth rate, etc. Those structural and functional changes in the skin due to skin aging facilitate geriatric skin diseases. In this article, mechanism of skin aging as well as manifestations of skin aging and some common skin diseases will be summarized.

## 1. Introduction

Skin is the largest organ of the body and serves crucial functions. There are three layers of skin, including epidermis, dermis and hypodermis. Epidermis forms the barrier to prevent outside antigen and other substances from passing through and also plays an important role in the immune system and combatting infections. Beneath epidermis, dermis forms a main interface to vasculature and nervous system in the skin, which provides nutrition, recirculating cells and cutaneous sensation [1]. The deepest layer, hypodermis (or subcutaneous tissue), contains a higher proportion of adipocytes and less collagen compared to the other layers.

Like other organs, skin undergoes biological aging, which is categorized as either endogenous or exogenous. Endogenous aging involves a series of physiological changes that gradually occur over time and are influenced by genes and hormones. In contrast, extrinsic aging (or exogenous aging), also known as photoaging, involves significant structural and functional changes due to external factors. This opinion will give some brief summaries about the cytological changes and clinical presentations according to skin aging as well as some common skin disorders in elderly populations.

## 2. Intrinsic and Extrinsic Aging

### 2.1. Intrinsic Aging

Like other organs, skin suffers progressive morphological and physiological decline with aging [2]. The mechanisms of intrinsic aging include the actions of reactive oxygen species (ROS), mtDNA mutations, telomere shortening, as well as hormonal changes [3]. In addition, several gene mutations have been correlated with the aging phenotype. This can be characterized by unblemished, dryer, paler, less elastic skin and wrinkles. Additional subcutaneous changes can occur within the tissue itself, via reductions in dermal mast cells, fibroblasts, collagen production, flattening of dermal–epidermal junction and loss of rete ridges.

### 2.2. Extrinsic Aging

For decades, extrinsic aging has been known to result from chronic exposure to the environment, especially solar radiation. Structural and functional changes in the skin due to sun exposure can be defined as “photoaging”. Moreover, other factors contributed to extrinsic aging, including tobacco smoking, diets, exposure to chemical substances, air pollution, etc. Extrinsic aging leads to the production of ROS, ultimately causing DNA damage and cellular dysfunction. In a study conducted in Germany, Andrea Vierkötter et al. have assessed the influence of air pollution on skin aging in 400 Caucasian women aged 70–80 years. The impact of air pollution on skin aging was analyzed by linear and logistic regression and adjusted for potential confounding variables. Air pollution exposure was significantly correlated to extrinsic skin aging signs, in particular to pigment spots and, to a lesser extent, wrinkles [4]. Moreover, two cross-sectional studies conducted in China also showed that cooking with solid fuels was significantly associated with a 5–8% more severe wrinkle appearance on face and 74% increased risk of having fine wrinkles on back of hands in both studies combined, regardless of age and other influences on skin aging [5].

Unlike intrinsic aging, extrinsic aging could be prevented and managed. Clinical symptoms of extrinsic aging include deep wrinkles, reducing elasticity, roughness, fragility, pigmentation disorders and telangiectasia (Table 1).

## 3. Structural and Functional Age-Related Skin Changes

**Figure 1:**
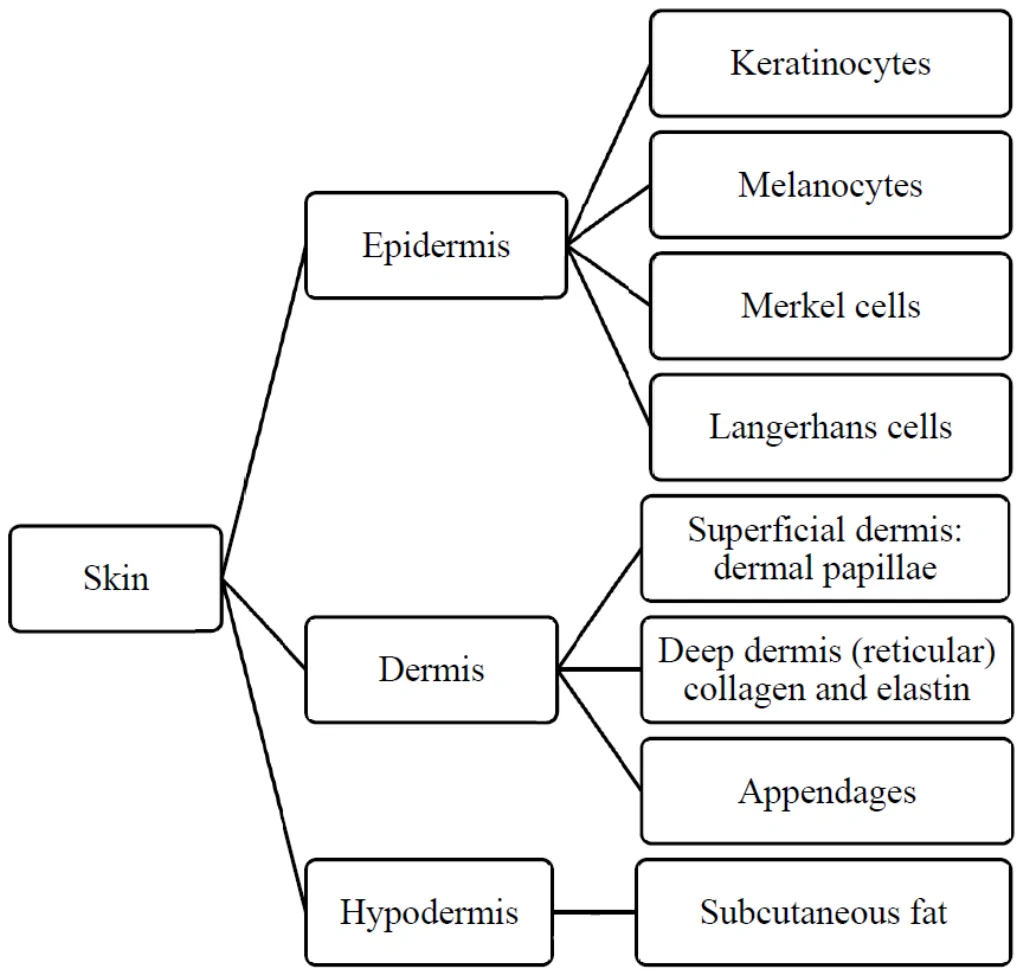
Layers of the skin and related cells.

Layers of the skin and related cells had been shown in Figure 1. and will be discussed in detailed. 

### 3.1. Epidermis

Between the third and eighth decade of life, the epidermal turnover rate reduces by about 30 to 50 percent when compared to younger age, resulting in reduced wound healing. During this period, there is also the thinning of the epidermis of approximately 10% to 50%. The spinous cell layer appears to be impacted the most by epidermal atrophy, whereas the stratum corneum and stratum granulosum are largely unaffected [6]. These histological changes manifest clinically as increased skin fragility and greater susceptibility to trauma and blister formation.

Although the average thickness of stratum corneum does not change with age, skin aging is more sensitive to irritated agents and xerosis. Skin aging also affects the permeability of drugs.

Decreased lipid enzyme functions and bilayer lipids in the stratum corneum, coinciding with reduced number of filaggrin, result in xerosis and desquamation of the skin. Because senescent cells are resistant to apoptosis, they may accumulate DNA mutations and protein damage over time. The buildup of senescent keratinocytes over time may provide the mechanistic link between aging and epidermal carcinogenesis [7].

### 3.2. Dermis

Biochemical changes in collagen—the main ingredient of the dermis and supportive structure of skin—may account for skin aging characteristics, including increased rigidity and impaired wound healing.

Skin aging involves extrinsic aging, mainly by UV exposure synergistically with intrinsic augment ROS production and retard ROS removal by antioxidants. ROS acts as the main factor contributing to aging skin not only in dermis but also in other organs of the body. Excessive ROS in fibroblasts can activate several signaling pathways leading to the accumulation of senescent fibroblasts, induction of chronic inflammation and disruption of extracellular matrix homeostasis [8,9,10].

Senescent dermal fibroblasts also play an important role in skin aging. Like other senescent cells, senescent dermal fibroblasts experience proliferation arrest, so that they remain viable and persist in the stroma due to their lack of recognized features to be captured by the immune system and apoptosis. Moreover, senescent cells can spread cell to cell by information transportation mechanisms, resulting in fueling the process of aging skin [11]. Collagen type I and III are reduced during intrinsic and extrinsic aging. Collagen production decreases over time, leading to fragment collagen, decreasing the physical tension of fibroblasts. Metalloproteinase activity in dermis contributes to environmental factors supporting collagen rupture. Increase in degradation and decrease in collagen production happen as a consequence of homeostatic imbalance.

Elastin, a component of dermis, takes part in elasticity and can change due to aging. Photoaging is represented by the replacement of normal elastic fibers with disordered elastotic material including degraded elastic fibers, tropoelastin, and fibrillin, which is localized near the dermal–epidermal junction [12]. This solar elastosis happens even in sun-protected areas in people older than 70 years. Elastin fibers reduce in number and diameter, become fragile and increase crosslink and calcification. These abnormalities translate into functional impairment and elastosis.

Skin aging not only affects collagen and elastin, but also the ground substance of skin, marked with proteoglycans and glycosaminoglycans, leading to reduced interaction between protein and water. Although glycosaminoglycans increase in aging, their deposition in abnormal elastotic form prevents water attraction. While intrinsic aging reduces hyaluronic acid gradually due to decreased secretion or extractability, changes in the ground substances of extrinsic aging make skin dehydrated and lose their strength.

### 3.3. Skin Appendages

Skin appendages include nails, hair, sebaceous glands, eccrine (sweat) glands, and apocrine glands. They have the following two distinct components: superficial and deeper components in dermis. They are responsible for sensation, temperature sensation, excretion, perspiration and thermoregulation.

#### 3.3.1. Hair

Because of hormonal effects overtime, there are changes in overall hair density and texture.

Hair follicles reduce in number in the older age groups compared to children [13]. This can be explained by the reduction in fibroblast number, circulation growth-factor levels, and microvessel density. Both men and women are affected by age-related alopecia. Senescent alopecia causes hair thinning, whereas androgenetic alopecia (or male-pattern hair loss) is a distinct entity that can occur at an earlier age.

In addition to hair loss, hair graying is another common age-related change. By the age of 60 years, nearly half of the population has at least 50% gray hair. Several recent advances in molecular biology and genetics have increased the understanding of the mechanism of hair graying. Hair color is provided by melanin produced by melanocytes and the development of hair graying relies on the decrease in melanin production and deposition within the hair shafts [14,15]. The reasons for the vulnerability of this specific melanocyte stem cell population are not completely understood but may be related to the high lifetime proliferative rate and relative sensitivity to oxidative stressors [16,17]. Besides genetic factors, other extrinsic factors can contribute to the graying of hair, including diet [18], vitamins, especially vitamin B12, smoking [19], stress and some diseases.

#### 3.3.2. Nail

Nail growth rate increases steadily until approximately the age of 25 and decreases afterward. Older people are at an increased risk of nail alterations, including normal age-related changes and disorders that more commonly affect this population. Changes during aging can be attributed to dysfunctional blood circulation at the distal extremities or extrinsic factors, although the exact underlying mechanism is still controversial. Age-related changes in the morphology of the nail plate include thickness, contour, surface and color [20,21]. In elderly individuals, nails are typically more brittle than in younger people. As a consequence of variation in lipid composition, older nails may also exhibit ridging. Nail consistency changes make the fingernail soften and weaken with age while toenails tend to thicken and harden [22]. Regarding discoloration of nails overtime, nails may turn yellow to gray discoloration with dull, pale, or opaque appearance, or even have longitudinal melanonychia.

#### 3.3.3. Cutaneous Glands and Nerves

There are the following three types of sweat glands in the human skin: eccrine, apoeccrine and apocrine sweat glands [23]. Eccrine sweat glands are the major type, which are widely distributed over the surface of human skin and are the main contributors to the regulation of body temperature. Cutaneous glands change dramatically over time in number and output of eccrine glands [24]. Aging worsens eccrine gland function, leading to the loss of heat tolerance and resulting in increased mortality due to heatstroke [25]. Aged skin also has reduced response to heat stimulation and stimuli from the nervous system. Sweat gland function has been reported to deteriorate with aging [26,27], and those are only local changes, not the central control of sweating changes. According to a study conducted by Tomonobu Ezure et al., results showed that there was no difference in the density and volume of sweat glands between young and old subjects [28]. Therefore, authors considered that age-dependent morphological changes might be related to functional change.

The number of sebaceous glands remains unchanged over time, while the function of sebaceous glands tends to initially increase with aging in the early stage, especially in light-exposed skin [29]. However, in the late stage of aging, due to the effects of hormonal changes and excessive light exposure, the sebaceous glands would atrophy [30]. Sebum excretion level is relatively low in children, starts to increase during puberty and decreases afterward. With aging, skin becomes dryer and is characterized by a lack of brightness of the skin surface, roughness, xerosis, desquamation and pruritus [31]. Older populations are more likely to have increased thermal pain threshold.

## 4. Geriatric Dermatoses

Geriatric dermatoses are challenging for physicians when it comes to diagnosis, management and follow-up. Since both intrinsic and extrinsic aging affect morphological and pathophysiological characteristics of older people, it is important for dermatologists to have a better understanding of the geriatric skin disorders and their specific characteristics. Some common geriatric dermatoses will be listed below.

### 4.1. Benign Skin Lesions

Benign proliferative growths commonly occur in aging skin. Skin discolorations, such as solar lentigines (also known as senile lentigines), are well-defined patches of hyperpigmentation that are associated with UV exposure. The reported average prevalence of solar lentigines ranges from 10% to 90% in older subjects [32]. The pathogenesis of solar lentigines is not completely understood; it might be due to repeated UV exposure resulting in increased melanin production and abnormal proliferation and differentiation of keratinocytes [33].

**Figure 2:**
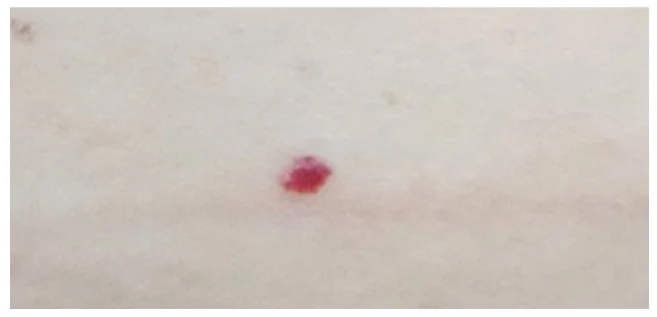
Senile angioma: erythematous to violaceous lobulated papules.

Senile angiomas (Figure 2), also known as cherry angiomas, are other benign skin lesions that affect the elderly population. On physical examination, lesions may have different appearances, ranging from a small red macule to a larger dome-topped papule. The typical color of the lesions is described as bright cherry red but may be more violaceous (purple) over time.

The prevalence of senile angiomas has been reported as 50% to 75% in older individuals [32].

### 4.2. Malignant Skin Lesions

Both benign and malignant neoplasms have been reported to have increased frequency in elderly subjects. In elderly patients, skin cancers represent approximately 40% of diagnosed malignant neoplasms, with increased risk of poor prognosis [34].

**Figure 3:**
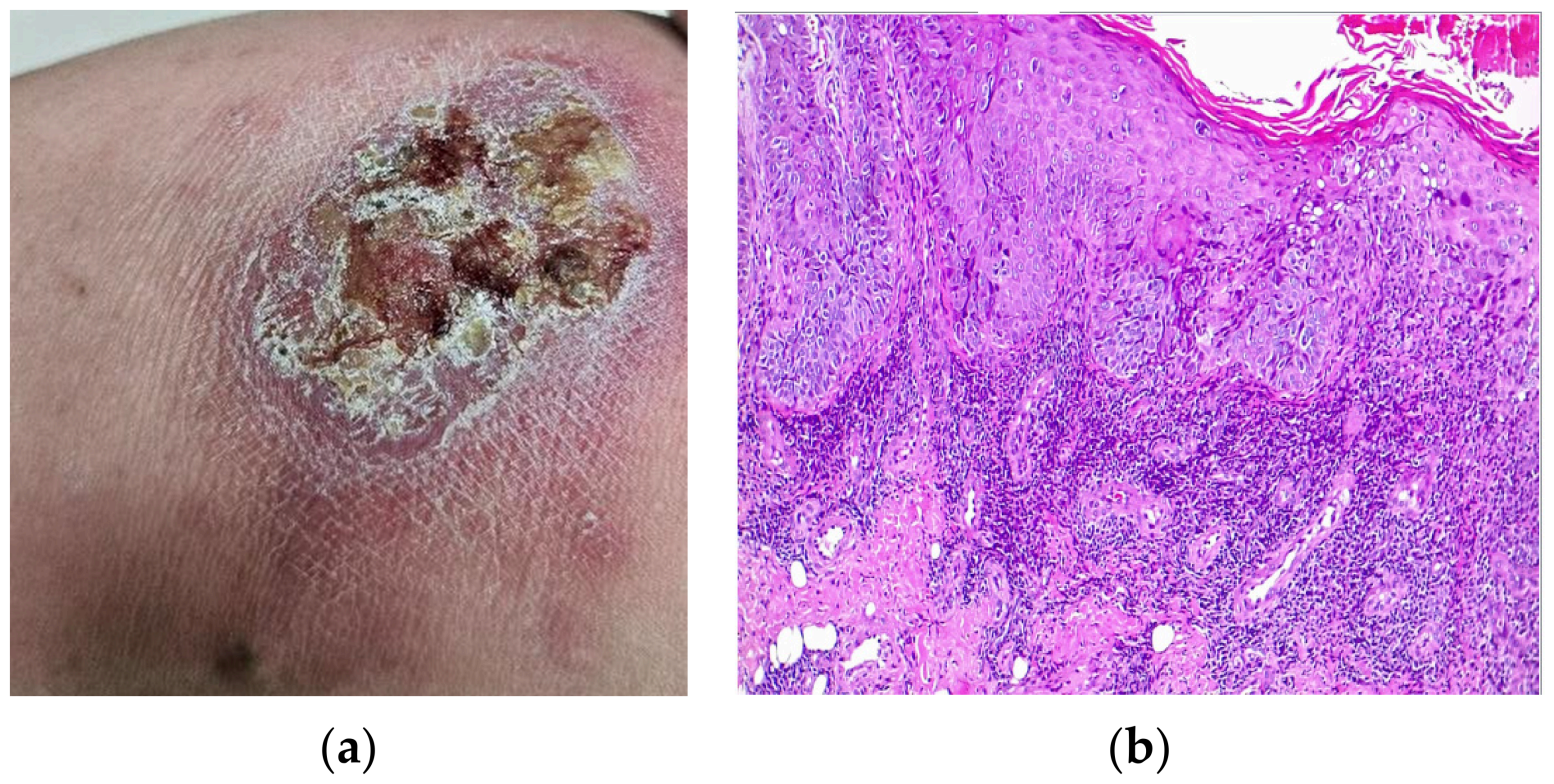
Squamous cell carcinoma in situ—Bowen disease in clinical (**a**) and histological (**b**) in an 55-year-old woman (source: Ho Chi Minh City Hospital of Dermato-Venereology).

An age-related reduction in cutaneous melanocyte density and skin barrier function results in more extensive penetration of UV light into the dermis of elderly individuals, thus causing more damage. Furthermore, patients at this age also experienced reduction in cell-mediated immunity, a function of Langerhans cells of the epidermis which are responsible for clinical expression of malignancy [35]. Although the vast majority of skin cancers are basal cell cancer, squamous cell cancers (Figure 3), melanoma and other types of carcinomas are associated with high morbidity and mortality.

### 4.3. Xerosis

Xerosis, or dry skin, is one of the most common skin disorders seen in the elderly. The causes of xerosis are multifactorial due to alterations of skin aging. Moreover, xerosis is also related to chronic renal failure, liver failure, lower-leg atherosclerosis, autoimmune diseases and hepatitis C infections [32]. Xerosis can be exacerbated by soap, hot water, and low humidity. Moisturizers are the mainstay treatment, and patients need to change their behavior and environment-related factors.

### 4.4. Pruritus

Pruritus or itchy skin is also another common complaint of elderly patients that significantly affects their quality of life [36]. Many skin disorders can be associated with pruritus, including xerosis or underlying systemic disease or malignancy. The exact pathogenesis is poorly understood. Age-related changes in the nerves leading to increased sensation thresholds have been suggested as a causation. Some other skin disorders such as scabies or tinea pedis are also typical causes of pruritus.

### 4.5. Infections

A variety of infections, including bacterial, viral, fungal and parasitic, may occur in the elderly population. Due to the presence of multiple comorbidities, decreased skin barrier function results in increased risk factors of skin infections in geriatric population. The clinical spectrum of skin infections can vary from mild infections to life-threatening diseases. More specifically, skin and soft tissue infections increased almost two-fold in older adults (>65 years of age) [37].

**Figure 4:**
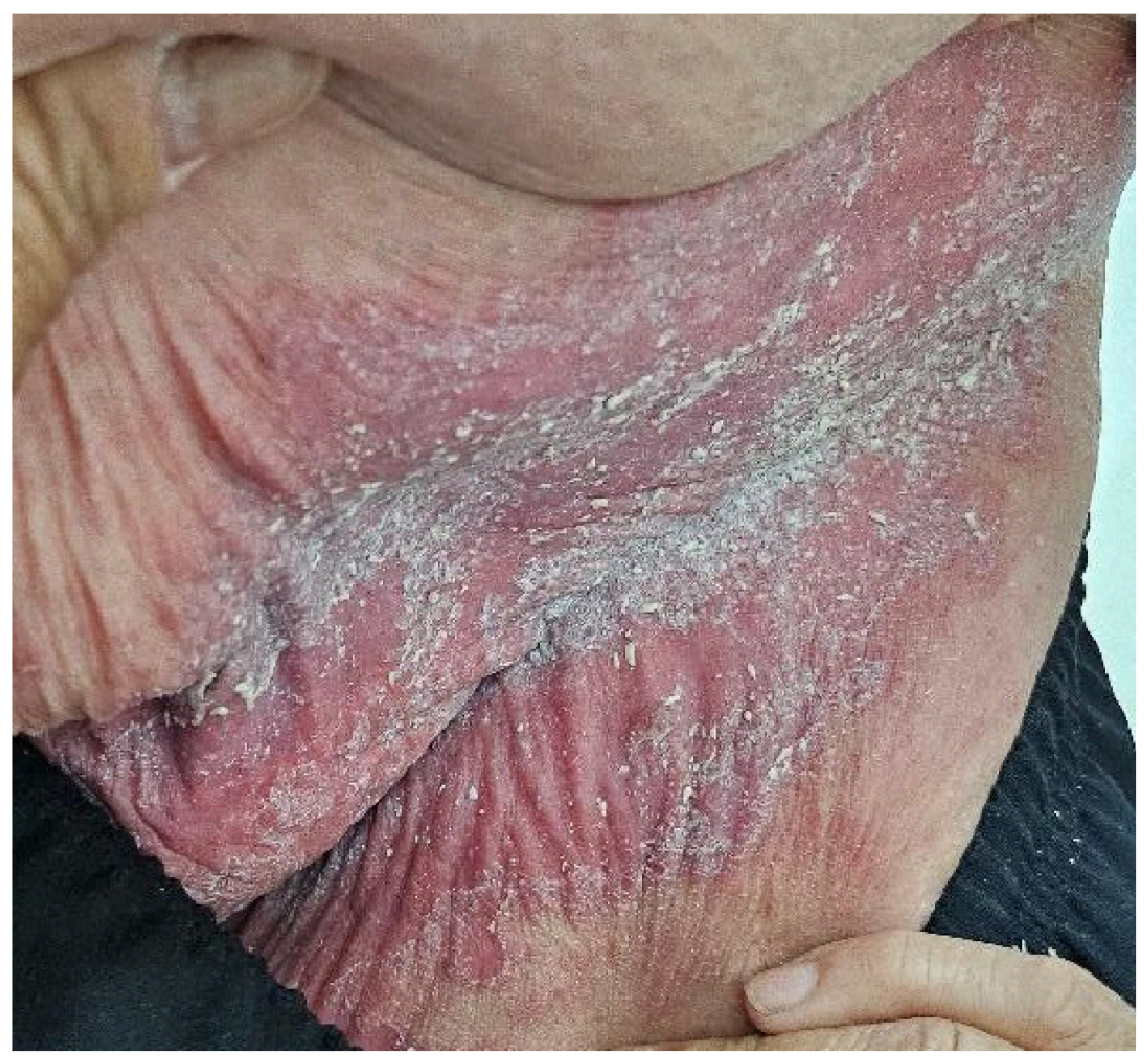
Candidiasis in an elderly person characterized by erythema, maceration and satellite lesions in axilla (source: Ho Chi Minh City Hospital of Dermato-Venereology).

Old age is also a risk factor for cutaneous fungal infections, age-associated decrease in immunity, vitamin deficiency, lack of motivation, peripheral vascular diseases and many comorbidities. There are the following three common types of cutaneous fungal infections: dermatophytes, yeast species, Candida (Figure 4), and Pytirosporum, another yeast species. Systemic antifungal treatments in this population should be cautioned because of their interactions with other comorbidities’ medications and decrease in the metabolic system.

Herpes zoster (HZ), commonly known as shingles, is a cutaneous viral disease that can occur in an estimated 20% to 30% of adults [38]. Risk of HZ increases with age and comorbidities [39]. Moreover, approximately up to 18% of patients with HZ develop postherpetic neuralgia (PHN), which can affect quality of life a lot [40].

Scabies and pediculosis are the most clinically significant parasitic infections in the geriatric population. The infection is caused by close and prolonged contact with infected patients. Early detection and treatment will help to limit the spread of the infestation.

### 4.6. Bullous Pemphigoid

Bullous pemphigoid is an autoimmune blistering disorder that commonly occurs in patients older than 60. While the clinical presentation of bullous pemphigoid is broad, it usually presents with tense bullae and intense generalized pruritus. Diagnosis of the bullous pemphigoid relies on the clinical characteristics and laboratory tests. Histology with indirect and direct immunofluorescence is necessary to confirm the diagnosis. A systemic review and meta-analysis published in 2022 demonstrated that advanced age, positive bullous pemphigoid 180 antibody, poor general condition, concomitant dementia, stroke, heart disease and diabetes mellitus were the factors that can influence the poor prognosis for mortality in patients with bullous pemphigoid [41].

### 4.7. Drug Eruptions

Elderly patients are likely to be on several medications because of comorbidities leading to high incidence of drug eruptions. The incidence of drug eruptions in the general population is reported to be 10–30% of all reported adverse drug reactions [42]. The clinical symptoms of drug eruptions can vary from the most common type—exanthematic eruptions fix drug eruption, erythema multiforme—to more serious types—Stevens–Johnson syndrome or toxic epidermal necrolysis. Thus, appropriate consideration and strict follow-up should be given for the geriatric population.

## 5. Conclusions

In conclusion, there are many changes related to skin aging including epidermis, dermis and appendages. These changes result in hypersensitivity to some geriatric skin disorders. Moreover, understanding physiologic and pathophysiological characteristics and concomitant medications will help dermatologists in better diagnosis and management in these specific populations. Further research into the molecular mechanisms underlying skin aging and the identification of novel biomarkers opens promises to improve the health and quality of life of older individuals.

## Figures and Tables

**Table 1:** Histologic and clinical presentations of skin aging.

Histological Presentations	Clinical Presentations
Epidermal thinning	Thinning of skin, roughness Xerosis, wound healing impairment
Loss of rete ridges	Fine and deep wrinkles, decreased elasticity, fragility
Decreased number of fibroblasts, collagen and elastin fibers	Laxity Dyspigmentation
Reduced amount of extracellular matrix	Solar elastosis Multiple telangiectasia, pallor

## Data Availability

No new data were created or analyzed in this study. Data sharing is not applicable to this article.

## References

[1] Garza L. (2019). Structure and Function of Skin. Fitzpatrick’s Dermatology.

[2] Assaf H., Adly M.A., Hussein M.R., Farage M.A., Miller K.W., Maibach H.I. (2010). Aging and Intrinsic Aging: Pathogenesis and Manifestations. Textbook of Aging Skin.

[3] Tobin D.J. (2017). Introduction to skin aging. J. Tissue Viability.

[4] Vierkötter A., Schikowski T., Ranft U., Sugiri D., Matsui M., Krämer U., Krutmann J. (2010). Airborne particle exposure and extrinsic skin aging. J. Investig. Dermatol..

[5] Li M., Vierkötter A., Schikowski T., Hüls A., Ding A., Matsui M.S., Deng B., Ma C., Ren A., Zhang J. (2015). Epidemiological evidence that indoor air pollution from cooking with solid fuels accelerates skin aging in Chinese women. J. Dermatol. Sci..

[6] Michelle L., Kerns A.L.C.S.K. (2019). Skin Aging. Skin Changes Across the Span of Life.

[7] Choi E.-H., Man M.-Q., Xu P., Xin S., Liu Z., Crumrine D.A., Jiang Y.J., Fluhr J.W., Feingold K.R., Elias P.M. (2007). Stratum corneum acidification is impaired in moderately aged human and murine skin. J. Investig. Dermatol..

[8] Ansary T.M., Hossain M.R., Kamiya K., Komine M., Ohtsuki M. (2021). Inflammatory Molecules Associated with Ultraviolet Radiation-Mediated Skin Aging. Int. J. Mol. Sci..

[9] Chen Q., Zhang H., Yang Y., Zhang S., Wang J., Zhang D., Yu H. (2022). Metformin Attenuates UVA-Induced Skin Photoaging by Suppressing Mitophagy and the PI3K/AKT/mTOR Pathway. Int. J. Mol. Sci..

[10] Gu Y., Han J., Jiang C., Zhang Y. (2020). Biomarkers, oxidative stress and autophagy in skin aging. Ageing Res. Rev..

[11] da Silva P.F.L., Ogrodnik M., Kucheryavenko O., Glibert J., Miwa S., Cameron K., Ishaq A., Saretzki G., Nagaraja-Grellscheid S., Nelson G. (2019). The bystander effect contributes to the accumulation of senescent cells in vivo. Aging Cell.

[12] Yaar M., Gilchrest B.A. (2007). Photoageing: Mechanism, prevention and therapy. Br. J. Dermatol..

[13] Arnal-Forné M., Molina-García T., Ortega M., Marcos-Garcés V., Molina P., Ferrández-Izquierdo A., Sepulveda P., Bodí V., Ríos-Navarro C., Ruiz-Saurí A. (2024). Changes in human skin composition due to intrinsic aging: A histologic and morphometric study. Histochem. Cell Biol..

[14] Liao C.-P., Booker R.C., Morrison S.J., Le L.Q. (2017). Identification of hair shaft progenitors that create a niche for hair pigmentation. Genes Dev..

[15] Tobin D., Paus R. (2001). Graying: Gerontobiology of the hair follicle pigmentary unit. Exp. Gerontol..

[16] Kauser S., Westgate G.E., Green M.R., Tobin D.J. (2011). Human hair follicle and epidermal melanocytes exhibit striking differences in their aging profile which involves catalase. J. Investig. Dermatol..

[17] Nishimura E.K., Granter S.R., Fisher D.E. (2005). Mechanisms of hair graying: Incomplete melanocyte stem cell maintenance in the niche. Science.

[18] Acer E., Erdoğan H.K., Iğrek A., Parlak H., Saraçoğlu Z.N., Bilgin M. (2019). Relationship between diet, atopy, family history, and premature hair graying. J. Cosmet. Dermatol..

[19] Poonia K., Bhalla M. (2024). Premature Graying of Hair: A Comprehensive Review and Recent Insights. Indian Dermatol. Online J..

[20] Cohen P.R., Scher R.K. (1992). Geriatric nail disorders: Diagnosis and treatment. J. Am. Acad. Dermatol..

[21] Singh G., Haneef N.S., A U. (2005). Nail changes and disorders among the elderly. Indian J. Dermatol. Venereol. Leprol..

[22] Richert B. (2019). The Aging Nail and Related Disorders. Baran & Dawber’s Diseases of the Nails and Their Management.

[23] Wilke K., Martin A., Terstegen L., Biel S.S. (2007). A short history of sweat gland biology. Int. J. Cosmet. Sci..

[24] Oberste-Lehn H. (1966). The number of sweat glands and aging in man. Arch. Klin. Exp. Dermatol..

[25] Rittié L., Fisher G.J. (2015). Natural and sun-induced aging of human skin. Cold Spring Harb. Perspect. Med..

[26] Larose J., Boulay P., Sigal R.J., Wright H.E., Kenny G.P. (2013). Age-related decrements in heat dissipation during physical activity occur as early as the age of 40. PLoS ONE.

[27] Inoue Y., Kuwahara T., Araki T. (2004). Maturation- and aging-related changes in heat loss effector function. J. Physiol. Anthropol. Appl. Hum. Sci..

[28] Ezure T., Amano S., Matsuzaki K. (2021). Aging-related shift of eccrine sweat glands toward the skin surface due to tangling and rotation of the secretory ducts revealed by digital 3D skin reconstruction. Ski. Res. Technol..

[29] Zouboulis C.C., Picardo M., Ju Q., Kurokawa I., Törőcsik D., Bíró T., Schneider M.R. (2016). Beyond acne: Current aspects of sebaceous gland biology and function. Rev. Endocr. Metab. Disord..

[30] Hou X., Wei Z., Zouboulis C.C., Ju Q. (2022). Aging in the sebaceous gland. Front. Cell Dev. Biol..

[31] Balin A.K., Pratt L.A. (1989). Physiological consequences of human skin aging. Cutis.

[32] Reszke R., Pełka D., Walasek A., Machaj Z., Reich A. (2015). Skin disorders in elderly subjects. Int. J. Dermatol..

[33] Aoki H., Moro O., Tagami H., Kishimoto J. (2007). Gene expression profiling analysis of solar lentigo in relation to immunohistochemical characteristics. Br. J. Dermatol..

[34] Syrigos K.N., Tzannou I., Katirtzoglou N., Georgiou E. (2005). Skin cancer in the elderly. In Vivo.

[35] Swift M.E., Burns A.L., Gray K.L., DiPietro L.A. (2001). Age-related alterations in the inflammatory response to dermal injury. J. Investig. Dermatol..

[36] Kini S.P., DeLong L.K., Veledar E., McKenzie-Brown A.M., Schaufele M., Chen S.C. (2011). The impact of pruritus on quality of life: The skin equivalent of pain. Arch. Dermatol..

[37] Tun K., Shurko J.F., Ryan L., Lee G.C. (2018). Age-based health and economic burden of skin and soft tissue infections in the United States, 2000 and 2012. PLoS ONE.

[38] van Oorschot D., Vroling H., Bunge E., Diaz-Decaro J., Curran D., Yawn B. (2021). A systematic literature review of herpes zoster incidence worldwide. Hum. Vaccines Immunother..

[39] Thompson R.R., Kong C.L., Porco T.C., Kim E., Ebert C.D., Acharya N.R. (2021). Herpes Zoster and Postherpetic Neuralgia: Changing Incidence Rates From 1994 to 2018 in the United States. Clin. Infect. Dis..

[40] Yawn B.P., Saddier P., Wollan P.C., Sauver J.L.S., Kurland M.J., Sy L.S. (2007). A population-based study of the incidence and complication rates of herpes zoster before zoster vaccine introduction. Mayo Clin. Proc..

[41] Chen X., Zhang Y., Luo Z., Wu Y., Niu T., Zheng J., Xie Y. (2022). Prognostic factors for mortality in bullous pemphigoid: A systematic review and meta-analysis. PLoS ONE.

[42] Naldi L., Conforti A., Venegoni M., Troncon M.G., Caputi A., Ghiotto E., Cocci A., Moretti U., Velo G., Leone R. (1999). Cutaneous reactions to drugs. An analysis of spontaneous reports in four Italian regions. Br. J. Clin. Pharmacol..

